# Prolonged ovarian hormone deprivation alters the effects of 17β-estradiol on microRNA expression in the aged female rat hypothalamus

**DOI:** 10.18632/oncotarget.5433

**Published:** 2015-10-09

**Authors:** Yathindar S. Rao, Cody L. Shults, Elena Pinceti, Toni R. Pak

**Affiliations:** ^1^ Department of Cell and Molecular Physiology, Stritch School of Medicine, Loyola University Chicago, Maywood, IL 60153, USA

**Keywords:** microRNA, estrogen, hypothalamus, aging, Gerotarget

## Abstract

Administration of 17β-estradiol (E_2_) has beneficial effects on cognitive function in peri- but not post-menopausal women, yet the molecular mechanisms underlying age-related changes in E_2_ action remain unclear. We propose that there is a biological switch in E_2_ action that occurs coincident with age and length of time after ovarian hormone depletion, and we hypothesized that age-dependent regulation of microRNAs (miRNAs) could be the molecular basis for that switch. Previously we showed that miRNAs are regulated by E_2_ in young compared to aged female rats. Here we tested whether increasing lengths of ovarian hormone deprivation in aged females altered E_2_ regulation of these mature miRNAs. In addition, we determined where along the miRNA biogenesis pathway E_2_ exerted its effects. Our results showed that age and increased lengths of ovarian hormone deprivation abolished the ability of E_2_ to regulate mature miRNA expression in the brain. Further, we show that E_2_ acted at specific points along the miRNA biogenesis pathway.

## INTRODUCTION

The average life expectancy has increased over the past century due to better living conditions and medical advancements [1]. Women in particular are living longer, yet the age of menopause has remained constant at approximately 50 years of age. This results in most women living nearly a third of their lives without high levels of circulating estrogens. Risk factors for many diseases, such as cognitive and mood disorders, increase in women following menopause [2, 3, 4] and these risk factors have been correlated with the sharp decline in circulating levels of estrogens. Hormone therapy (HT) is often prescribed for women to mitigate many of the adverse symptoms associated with menopause, based primarily on evidence from animal models demonstrating that estrogens are anxiolytic and increase cognitive function [5, 6, 7, 8, 9, 10]. Data from studies in postmenopausal women, however, are less convincing that HT is beneficial. For instance, results from the large-scale clinical trial (Women's Health Initiative (WHI)) showed that there was a temporal discrepancy in the beneficial efficacy of HT [3, 4, 11, 12, 13, 14, 15, 16, 17]. The beneficial effects were largely dependent on the length of time following menopause onset that women received HT, with younger women realizing the greatest benefits. Follow up studies have since revealed that timing of HT is critical, both for maximum benefits, as well as minimal detrimental effects [11, 12, 13, 14, 15, 16]. However, the molecular mechanisms underlying these age-dependent effects of estrogens are unknown.

Estrogens regulate gene transcription primarily through binding to estrogen receptors (ERs), which then act as transcription factors on gene promoters. However, estrogens have also been shown to regulate gene expression at the post-transcriptional level through microRNAs (miRNAs). miRNAs are a class of small non-coding RNAs that regulate gene expression via translational repression of target mRNAs [18, 19], thereby decreasing the protein products of those genes. The canonical miRNA biogenesis pathway reveals several potential regulatory points for estrogens. First, miRNAs are transcribed from the genome raising the possibility that estrogen receptors mediate miRNA transcription. Second, miRNAs are regulated post-transcriptionally by a series of RNase III enzymes, which sequentially cleave the primary transcript (pri-miRNA) into shorter fragments (i.e. pre-miRNA), and these enzymes act in conjunction with several cofactor proteins, some of which are regulated by estrogens [20, 21, 22, 23, 24, 25, 26, 27]. Finally, the mature 22nt miRNA duplex dissociates such that one strand (guide strand) is loaded onto an argonaute (AGO) protein, thereby forming the RNA-induced silencing complex (miRISC). The guide strand is largely protected from degradation due to its association with target mRNA and AGO, yet the passenger strand is typically degraded. Several studies have shown that miRNAs are subject to post-transcriptional modifications, which can affect their stability. For instance, poly(A) polymerase associated domain 4 (PAPD4) is a nucleotidyltransferase that adenylates the 3′ end of miRNAs [28, 29]; this modification to the miRNA can increase its stability and alter its ability to bind to the RISC complex [28, 29]. Ultimately, miRNAs are degraded by at least one known exonuclease, 5′-3′ exoribonuclease 2 (XRN2) [30]. To our knowledge, there have been no studies to date demonstrating whether estrogens regulate any proteins that are involved in miRNA stabilization pathways.

In the brain miRNAs display both an age-and brain region-specific expression pattern, suggesting that they play critical roles in normal brain function [31, 32, 33, 34, 35, 36, 37]. Indeed previous studies have demonstrated that miRNAs are crucial for neuronal development and synaptic plasticity, and are also implicated in regulating affective behaviors [38, 39, 40, 41, 42, 43, 44, 45]. Previously, our laboratory demonstrated that the major circulating estrogen, 17β-estradiol (E_2_), regulates miRNAs in an age- and brain region-dependent manner in female rats, suggesting that there would be differential mRNA translation in those brain regions at varying ages due to E_2_ dependent miRNA regulation [46]. E_2_ exerts its effects through two estrogen receptors, ERα and ERβ. ERα and ERβ display brain region specific expression patterns, and also have dichotomous effects on brain function [47, 48]. Additionally, both receptors have been shown to regulate miRNA expression *in-vitro*, however it is not known whether one or both receptors are required for mediating E_2_-induced miRNA expression in the brain [49, 50].

We propose that there is a biological switch in estrogens’ actions that occurs coincident with age and length of time after ovarian hormone depletion, and we hypothesize that age dependent regulation of miRNAs could be the molecular basis for that switch. Our previous work showed that E_2_ differentially regulated miRNA expression in the brain of young (3 mo.) compared to aged (18 mo.) female rats. In this current study we extended those observations by investigating if longer periods of E_2_ deprivation in aged female rats altered the E_2_-dependent regulation of miRNAs we previously observed in the brain. In this paradigm female Fisher 344 rats (18 mo. old) were ovariectomized (OVX) and then given an acute E_2_ treatment at 1, 4, 8, or 12 weeks post-OVX. Additionally, we had a group of ovarian intact female rats that were sacrificed at the same age as the treated animals. We analyzed the expression of our previously identified E_2_ regulated mature miRNAs, as well as their intermediary biosynthetic products (pri-miRNA, pre-miRNA) in the hypothalamus. In addition, we quantified the expression of several miRNA processing proteins including drosha, DiGeorge syndrome critical region 8 (DGCR8), exportin-5 (XPO5), dicer, AGO2, XRN2, and PAPD4. Finally, we determined which ER mediates the E_2_-induced increase in miRNA expression observed at 1 wk. post-OVX in the aged female brain. Collectively our results show that extended deprivation of ovarian hormones markedly alters E_2_ regulation of mature miRNAs in the aged female hypothalamus, suggesting that there is a shift in how the brain responds to the re-introduction of E_2_ after prolonged periods of ovarian hormone deprivation.

## RESULTS

### Experiment 1: Expression of E_2_-responsive mature miRNAs in the hypothalamus of ovarian intact animals changes with age

**Figure 1:**
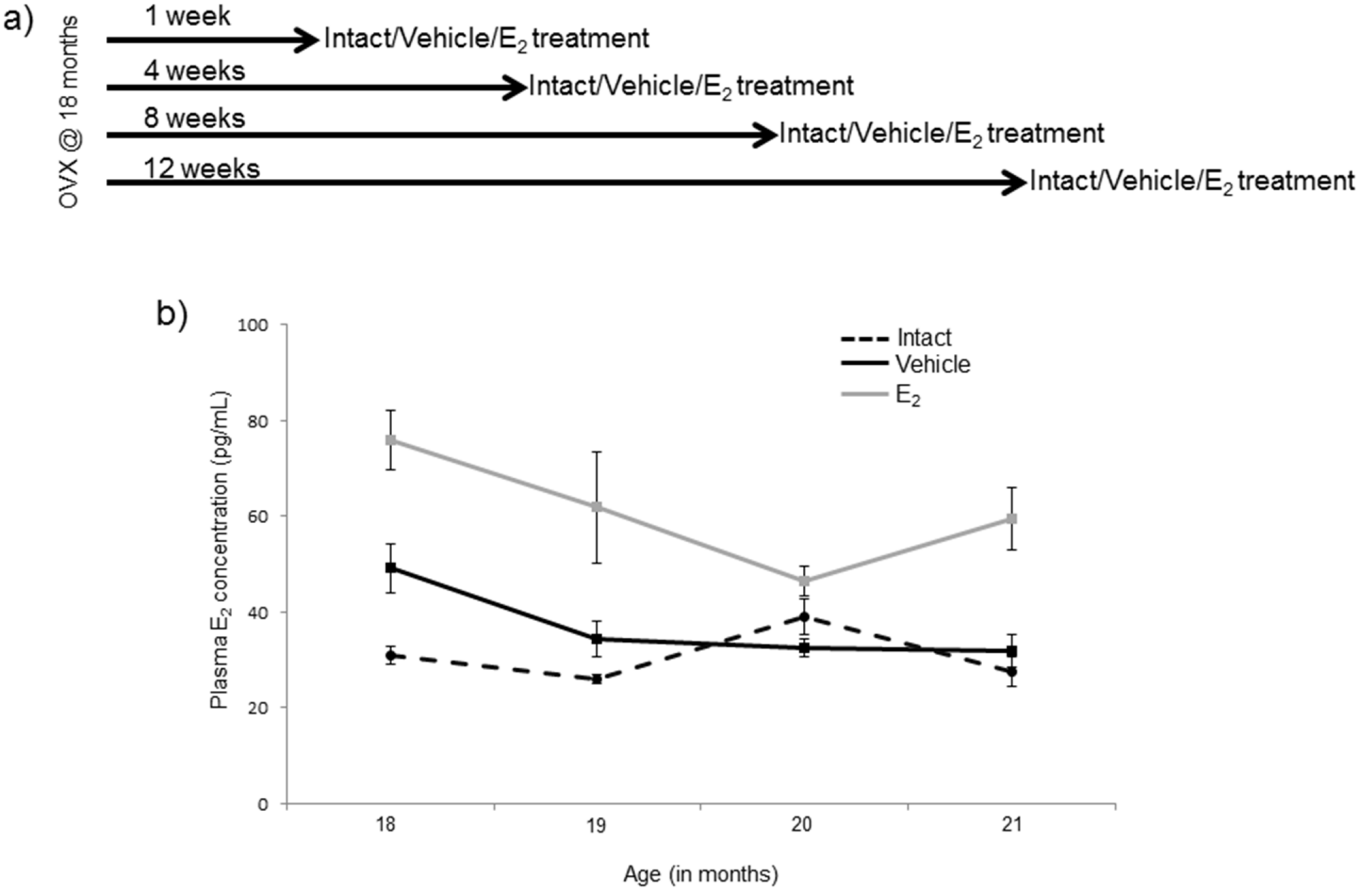
Diagram of ovarian hormone deprivation paradigm **a.** Schematic representation of the animal experimental paradigm. Fischer 344 female rats were obtained at 18 months of age and ovariectomized. Rats were given a subcutaneous injection once/day for 3 days 1, 4, 8, or 12 weeks post-OVX (*N* = 6/age/treatment). **b.** E_2_ plasma concentrations assayed by ELISA from blood samples taken 24 hours after the last injection of E_2_. Data displayed as mean ± SEM pg/mL.

**Figure 2:**
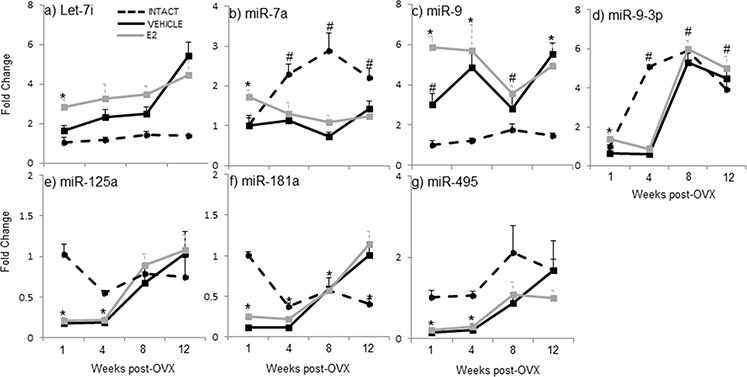
E_2_ regulation of mature miRNA expression in the hypothalamus after increasing lengths of ovarian hormone deprivation **a–g.** Mature miRNA expression was analyzed by real time qRT-PCR and displayed as mean ± SEM fold change as compared to 18 month old ovarian intact animals (*N* = 6/age/treatment). An * denotes a statistically significant effect of treatment within a time point.

**Figure 3:**
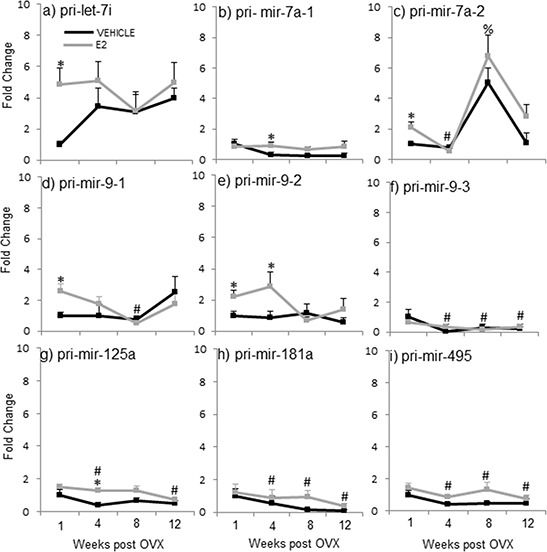
E_2_ regulation of the primary miRNA expression in the hypothalamus after increasing lengths of ovarian hormone deprivation **a–i.** Primary miRNA expression was analyzed by real time qPCR and displayed as mean ± SEM fold change as compared to 1 week vehicle treated animals (*N* = 6/age/treatment). An * denotes a statistically significant effect of treatment within a time point. Different symbols (#, %) denote a statistically significant difference across time points within a treatment group.

Our previous studies showed that E_2_ regulated a subset of mature miRNAs (let-7i, miR-7a, miR-9, miR-9–3p, miR-125, miR-181a, and miR-495) in an age- and brain-region dependent manner [46]. Here, we observed that the expression of mature miRNAs in ovarian intact animals at varying advanced ages was different from both the OVX-vehicle and OVX-E_2_ treated animals, suggesting that ovarian factors other than E_2_ also regulate mature miRNA expression in aged female rats. Notably, the circulating E_2_ levels were equivalent between ovarian intact and OVX+veh treated animals (Figure 1). A post-hoc analysis revealed a statistically different expression profile at the one-week post-OVX time point (i.e. 18 mo. old) for miR-9, miR-125a, miR-181a, and miR-495 in ovarian intact animals compared to OVX+veh treated animals. Further, there was a statistically significant difference in the expression of mature miR-7a, miR-9a-3p, and miR-181a between ovarian intact and OVX+veh treated animals at later time points (i.e. 19, 20, and 21 months). Specifically, let-7i: levels were unchanged across all time points in animals that were ovarian intact (Figure 2a), yet there was a steady increase in let-7i over time in OVX+veh treated animals (Figure 2a). miR-7a: By contrast, miR-7a expression was similar between ovarian intact and OVX+veh treated animals at the one week time point (= 18 mo. old). The levels continued to increase with age in ovarian intact animals, but this result was not observed in either OVX group (Figure 2b). miR-9: Similar to let-7i, we observed no changes in miR-9 expression across all time points in ovarian intact animals (Figure 2c), but there was a significant increase over time in OVX+veh animals. miR-9-3p: Interestingly, miR-9-3p was similar in all groups at one week post OVX (i.e. 18 mo., Figure 3d), yet miR-9-3p expression significantly increased at 19 mo. in the ovarian intact groups and this increase was not apparent in the OVX groups until 20 mo. (8 weeks post-OVX) and 21 mo. (12 weeks post OVX). miR-125a: Ovarian intact animals had significantly higher levels of miR-125a expression at 18 mo. and 19 mo. time points, however all groups were similar after 20 and 21 mo. (Figure 2e). miR-181a: Ovarian intact animals had significantly higher levels of miR-181a expression at 18 mo. old compared to both OVX+veh and OVX+E_2_ groups (Figure 2f). These expression levels decreased with age, whereas an opposite trend was observed in the OVX animals and miR-181a expression was significantly higher in both OVX groups compared to ovarian intact animals by 21 mo. of age (Figure 2g). miR-495: Ovarian intact animals had consistently higher levels of mature miR-495 expression at all ages compared to OVX animals, although both OVX+veh and OVX+E_2_ groups showed significant increases after 8 and 12 weeks post-OVX (20 and 21 mo.). Taken together, these data underscore the importance of ovarian factors other than E_2_ in regulating mature miRNA expression in the aged female brain.

### Experiment 2: Treatment with E_2_ failed to regulate mature miRNA expression following prolonged periods of ovarian hormone deprivation

To further investigate the effects of E_2_-mediated regulation of these mature miRNAs, we developed an animal paradigm to directly test the timing hypothesis in aged female rats. Aged rats (18 mo., equivalent to 55 years old in human) were ovariectomized (OVX) to model surgically induced menopause and eliminate the source of all ovarian hormones. To specifically isolate the effects of E_2_ from all other ovarian hormones, OVX animals were treated with acute administration of E_2_ or vehicle control following varying lengths of time post-OVX: 1, 4, 8, or 12 weeks (Figure 1a). Our results demonstrated that there was a statistically significant interaction between treatment and length of ovarian hormone deprivation (Table 1). Most striking was the fact that E_2_ treatment significantly regulated the expression of these miRNAs at only one time point (i.e. one week post-OVX) and had no effect following prolonged periods of ovarian hormone deprivation. Specifically, E_2_ treatment significantly regulated the expression of mature miR-7a, miR-9, miR-9-3p, and miR-181a at 1 week post-OVX, which was consistent with our previously published data, [46], but not at any other time point (Figure 2a–2d, 2f), demonstrating a clear timing effect. Interestingly, E_2_ treatment also increased the expression of miR-9-3p compared to intact animals, but not OVX+veh treated animals (Figure 2d).

**Table 1:** 2 way-ANOVA analysis of mature miRNA expression in the hypothalamus

miRNA	INTERACTION
Let-7i	NO
miR-7a	YES (*F*(6,57) = 7.065, *p* < 0.0001)
miR-9	YES (*F*(6,57) = 4.824, *p* < 0.0001)
miR-9-3p	NO
miR-125a	YES (*F*(6,57) = 4.077, *p* = 0.002)
miR-181a	YES (*F*(6,57) = 14.832, *p* < 0.0001)
miR-495	YES (*F*(6,57) = 3.958, *p* = 0.002)

### Experiment 2: Mature miRNA expression levels do not correspond to the expression of their primary (pri-) and precursor (pre-) forms

#### Effects of E_2_ treatment on primary miRNA (pri-miR) expression levels in the hypothalamus following varying lengths of ovarian hormone deprivation

The data from these initial experiments were consistent with our previously published findings and demonstrated that E_2_ regulates mature miRNA expression following short-term, but not long-term, OVX. In order to determine the level of the miRNA biosynthetic pathway that E_2_ acts, we next analyzed the expression of the miRNA primary (pri-miR) and precursor (pre-miR) transcripts. A two-way ANOVA analysis revealed that there was no significant interaction between treatment and age for the pri- or pre- forms of these miRNAs, contrary to the results we observed for the mature miRNAs. Moreover, analysis of treatment within a single time point showed that E_2_, in general, had no effect on the transcription of most of the pri-miRNAs at any time point (Figure 3). However, there were a few exceptions. Specifically, comparison of treatment within a time point revealed that E_2_ significantly increased expression of the primary miRNA transcript of let-7i one-week post OVX, but not at any other time point (Figure 3a, gray line, *). Also, it is important to note that two of the mature miRNAs, miR-7a and miR-9, are transcribed from multiple chromosomes, allowing for unique regulation of biogenesis at each locus. Therefore, we designed primers corresponding to distinct primary sequences on each chromosome for miR-7a and miR-9, in order to account for possible differences between mature miRNA products derived from the different chromosomes. Interestingly, these data revealed a switch in E_2_ regulation of each chromosome depending on the age post-OVX. Specifically, E_2_ increased pri-miR-7a transcribed from chromosome 1 at 4 weeks post-OVX, but not any other time points (Figure 3b, pri-miR-7a-1, gray line, *). Meanwhile, E_2_ significantly increased pri-miR-7a transcribed from chromosome 17 at 1-week post-OVX (Figure 3c, pri-miR-7a-2, gray line, *). These data suggest that the mature miR-7a product was derived from different chromosomes depending on the time point. Interestingly, however, our data only showed a significant increase in mature miR-7a at the 1-week time point, which points to chromosome 17 as potentially more important than chromosome 1 for E_2_-regulated mature miR-7a expression. It is also important to note that miR-9 (guide strand) and miR-9-3p (passenger strand) are derived from the same primary and precursor transcripts, which are located on two different loci on chromosome 1 (pri-miR-9-1, pri-miR-9-2) and one locus on chromosome 2 (pri-miR-9-3). Our previously published data, and also replicated here in Experiment 2, showed that E_2_ significantly regulated both the guide and passenger strands of mature miR-9 [46]. Figure 3d–3f depicts results from pri-miR-9. Analysis of the primary miR-9 expression showed that E_2_ treatment significantly increased pri-mir-9-1 and pri-mir-9-2 at 1-week post OVX, as well as pri-mir-9-2 at 4 weeks post OVX (Figure 3d, 3e, gray line, *). By sharp contrast, E_2_ did not affect the expression levels of pri-mir-9-3 at any time point (Figure 3f, gray line, *). Finally, E_2_ had no effect on pri-miR-181a or pri-miR-495 at any time point (Figure 3h, 3i).

#### Effects of OVX+vehicle on primary miRNA (pri-miR) expression levels in the hypothalamus following varying lengths of OVX

One way ANOVA analyses across the deprivation time points showed that pri-mir-7a-2, pri-mir-9-1, pri-mir-125a, pri-mir-181a, and pri-mir-495 were all significantly altered by OVX alone, and in general, they were all decreased with age (Figure 3, black line, #). Specifically, expression levels of pri-mir-9-1 (Figure 3d, black line, #) were significantly decreased at 8 weeks post OVX, while pri-mir-125a expression was decreased at 12 weeks post OVX (Figure 3g, black line, #). Further, both primary transcripts for miR-181a and miR-495 were significantly decreased at 4, 8, and 12 weeks post OVX (Figure 3h–3i, black line, #). The only exception was for pri-mir-7a-2, which was significantly decreased at 4 weeks, but then increased at 8 weeks post OVX (Figure 3c, black line, #).

#### Effects of OVX+veh and OVX+E_2_ treatment on precursor miRNA (pre-miR) expression levels in the hypothalamus

**Figure 4:**
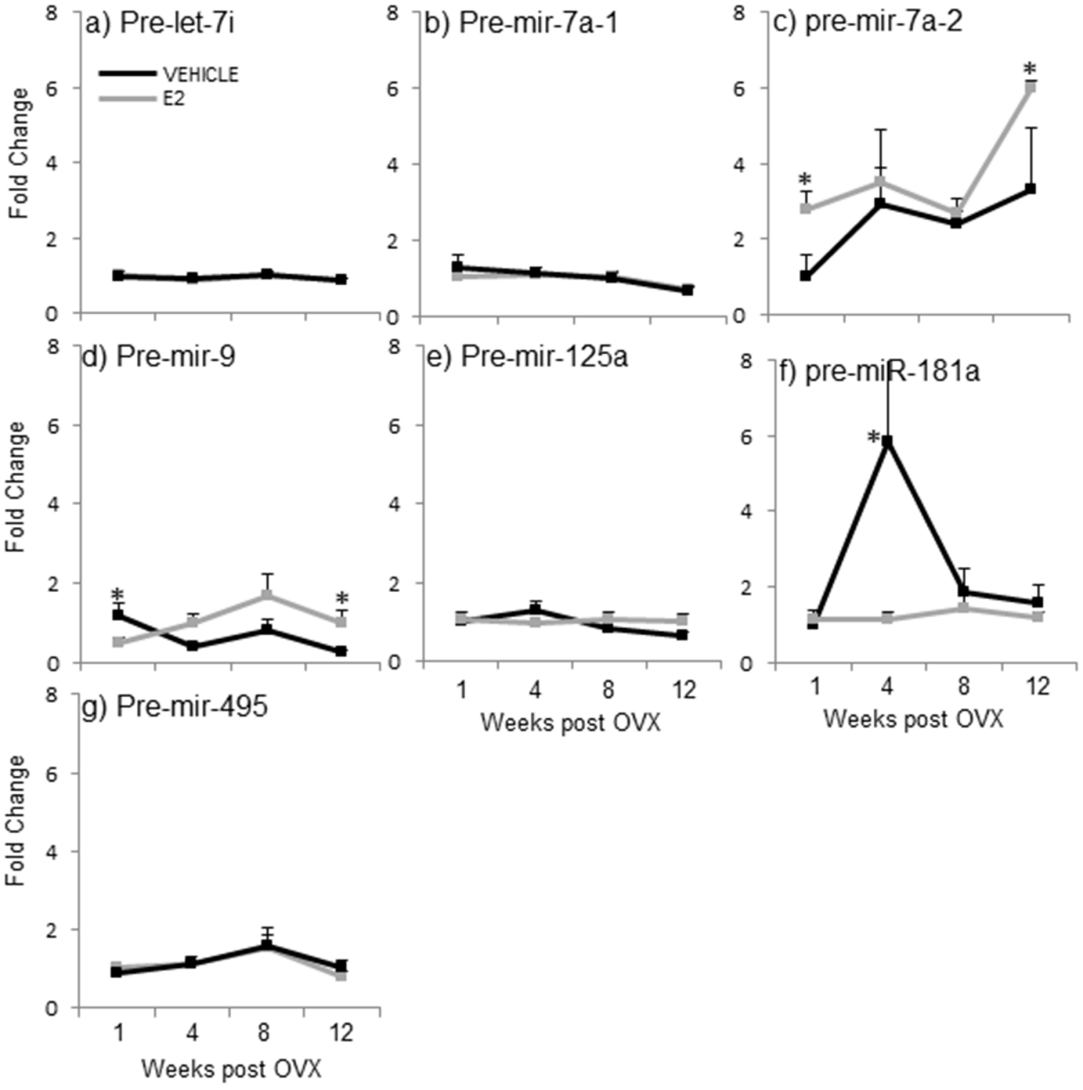
E_2_ regulation of the precursor miRNA expression in the hypothalamus after increasing lengths of ovarian hormone deprivation **a–g.** Precursor miRNA expression was analyzed by real time qPCR and displayed as mean ± SEM fold change as compared to 1 week vehicle treated animals (*N* = 6/age/treatment). An * denotes a statistically significant effect of E_2_ as compared vehicle treated animals within a time point.

A two-factor ANOVA analysis revealed that there was no significant interaction between age and treatment for any of the precursor miRNAs, again suggesting that the two factors are independent. Next, statistical analyses within each separate time point demonstrated that E_2_ treatment significantly altered the expression levels of 3 out of 7 pre-miRNAs tested when compared to vehicle-treated controls (Figure 4, gray line, *). Interestingly, E_2_ treatment significantly increased the expression of pre-miR-7a-2 at both 1 and 12 weeks post-OVX, but had no effect on pre-miR-7a-1 at any time point (Figure 4b, 4c, gray line, *). These results sharply contrast what was observed with the primary transcripts of these same miRNAs (see Figure 3b, 3c), suggesting that E_2_ acts differently at multiple levels of the miRNA biogenesis pathway. Similarly, the effects of E_2_ treatment were strikingly differently for pre-miR-9 (Figure 4d) compared to its effects on the primary form (pri-miR-9; see Figure 3d–3f). Specifically, E_2_ significantly decreased pre-mir-9 expression at 1 week post OVX, but then significantly increased its expression at 12 weeks post OVX (Figure 4d, gray line, *). While the primary transcript of miR-9 is transcribed from three different loci (hence, pri-miR-9-1, pri-miR-9-2, and pri-miR-9-3), the precursor hairpins generated from each primary transcript are too similar to be able to differentiate them using qRT-PCR. Therefore, it is possible that there were offsetting changes in each precursor form that might have obscured the final results. Interestingly, E_2_ completely abolished the steep age-related increase in pre-miR-181a observed at 4 weeks post-OVX (Figure 4f, gray line, *). These results were unexpected given the E_2_-induced increase in both mature and pri-miR-181a expression levels at varying time points (see Figures 2f and 3h). Finally, there were no effects of OVX alone on the expression levels for any of the precursor miRNAs tested (Figure 4, black lines).

### Experiment 2: E_2_ and age-related changes in mature miRNA expression are not explained by concomitant changes in the mRNA or protein levels of key components involved in miRNA biogenesis

**Figure 5:**
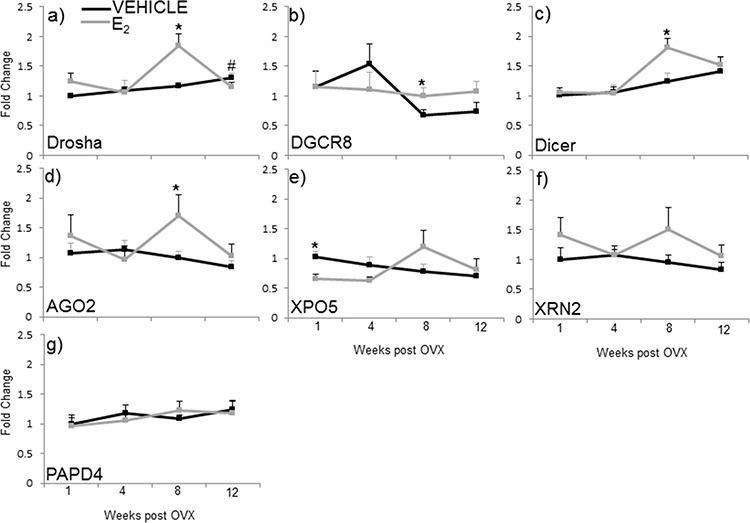
mRNA expression of miRNA biogenesis components in the hypothalamus after increasing lengths of ovarian hormone deprivation **a–g.** mRNA expression was analyzed by real time qPCR and displayed as mean ± SEM fold change as compared to 1 week vehicle treated animals (*N* = 6/age/treatment). An * denotes a statistically significant effect of treatment within a time point.

Our data show that E_2_ regulates miRNA expression at various levels along the miRNA biosynthetic pathway, raising the possibility that E_2_ could regulate the transcription (mRNA) and/or protein levels of critical components required for miRNA biosynthesis. Therefore, we analyzed the mRNA expression of the nuclear proteins drosha and DGCR8 (key components of the microprocessor complex), as well as cytoplasmic proteins dicer, AGO2, XPO5, XRN2, and PAPD4 in the hypothalamus of animals subjected to our ovarian hormone deprivation paradigm (Figure 5). The statistical analysis revealed that there was a significant interaction of treatment and age on the mRNA expression levels of drosha, but not any of the other genes tested (*F*(3,38) = 3.762, *p* = 0.019). Specifically, the post hoc analysis revealed that E_2_ treatment increased drosha mRNA expression at 8 weeks post OVX (Figure 5a). Statistical analyses within each time point revealed that E_2_ treatment also increased DGCR8, dicer, and AGO2 mRNA expression levels at 8 weeks post OVX (Figure 5b–5d). Conversely, E_2_ treatment significantly decreased XPO5 at 1-week post OVX (Figure 5e). We did not observe any significant changes in the mRNA expression XRN2 and PAPD4 between treatment groups at any time point (Figure 5f, 5g, gray line). Moreover, we did not observe changes due to OVX alone in the mRNA expression of these genes (black lines).

**Figure 6:**
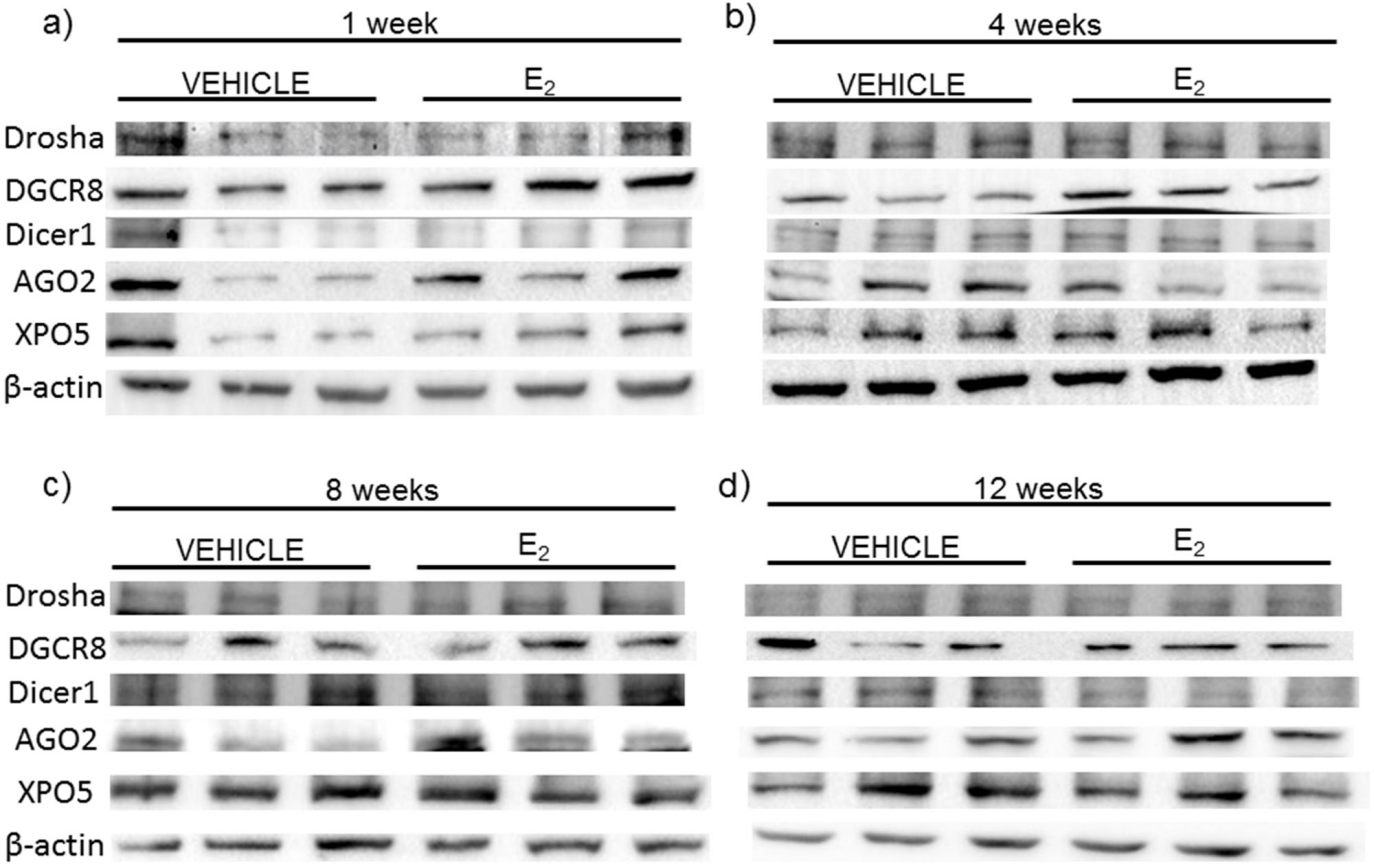
Protein expression of miRNA biogenesis components in the hypothalamus after increasing lengths of ovarian hormone deprivation **a–d.** Protein expression was analyzed by western blot analysis. 60 μg of total protein for each sample was run on 4–10% polyacrylamide gel. Blots were incubated with the indicated antibodies and visualized on Bio-Rad Chemidoc. Representative blots are shown (*N* = 3/treatment/age group).

**Figure 7:**
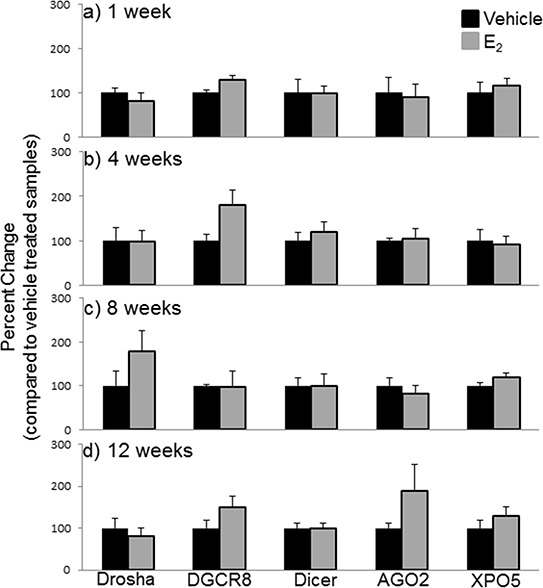
Protein expression of miRNA biogenesis components in the hypothalamus after increasing lengths of ovarian hormone deprivation **a–d.** Quantification of western blots described in figure 7 (*N* = 6/treatment/age group). Data are represented as mean ± SEM percentage as compared to vehicle treated animals. No statistical significance was observed between treatment groups.

Next, we compared the mRNA levels of drosha, DGCR8, dicer, AGO2, and XPO5 with their translated protein levels from the same animals (Figures 6, 7). Interestingly, there was no statistically significant effect of treatment or OVX on the protein levels of any of these genes, despite our observed statistically significant changes in mRNA levels. These data suggest that E_2_-mediated changes in mature miRNA expression levels cannot be explained by mRNA/protein changes in these key miRNA biosynthetic components.

### Experiment 3: ERβ mediates E_2_ effects on mature miRNA expression in the hypothalamus of aged female rats one-week post-OVX

**Figure 8:**
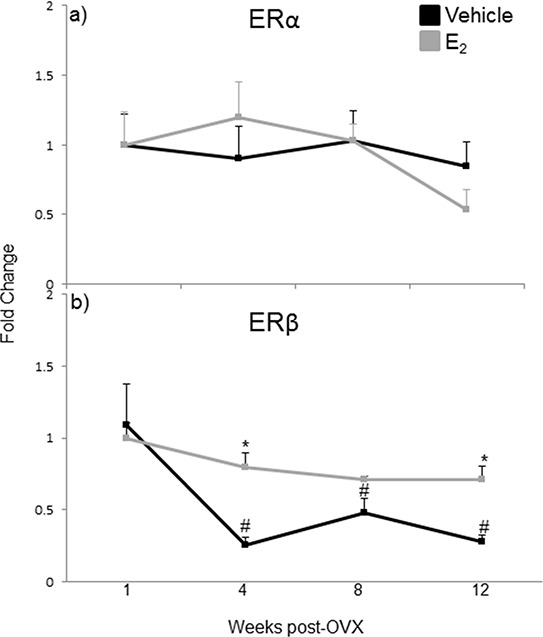
mRNA expression of estrogen receptor isoforms in the hypothalamus after increasing lengths of ovarian hormone deprivation **a–b.** mRNA expression of ERα (a) and ERβ (b) was analyzed by real time qRT-PCR and displayed as mean ± SEM fold change as compared to 1 week vehicle treated animals (*N* = 6/age/treatment. An * denotes a statistically significant effect of treatment within a time point within a treatment group. A # denotes a statistically significant difference across time points within a treatment group.

**Figure 9:**
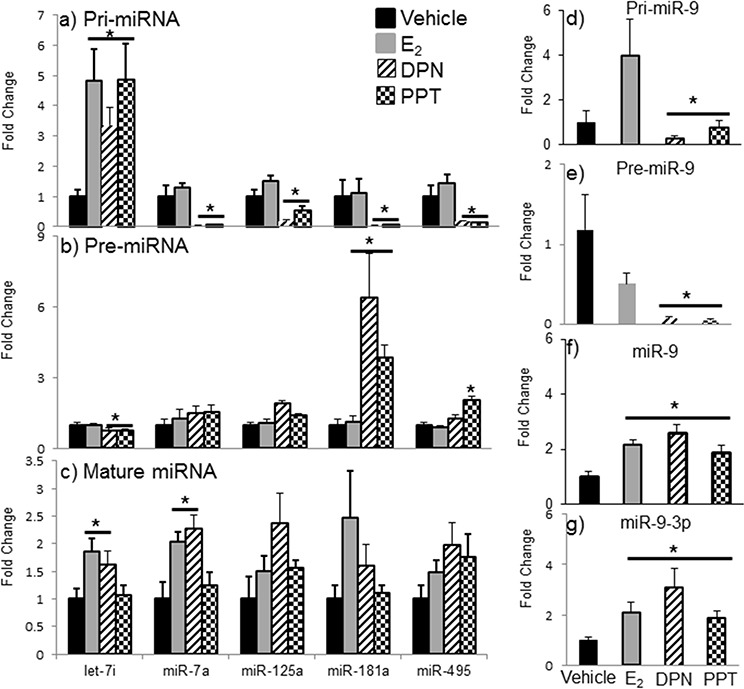
Effects of ERα and ERβ specific agonists on mature miRNA expression in the aged hypothalamus following 1 week of ovarian hormone deprivation Pri-miRNA **a, d.** pre-miRNA **b, e.** and mature miRNA **c, f, g.** were measured by qRT-PCR from animals administered vehicle, E_2_, DPN (ERβ agonist), or PPT (ERα agonist). Data are displayed as mean ± SEM fold change as compared to vehicle treated animals (*N* = 6/age/treatment). An * denotes a statistically significant effect of E_2_ as compared vehicle treated animals within a time point.

The effects of E_2_ are mediated primarily by two estrogen receptors, ERα and ERβ, and both have been shown to regulate miRNA expression in cancer cell lines [49, 50, 51]. To determine which receptor mediates the regulation of miRNA expression in the aging female brain, we first examined the expression levels of ERα and ERβ mRNA in our ovarian hormone deprivation paradigm. A two-way ANOVA analysis revealed that there was a statistically significant interaction between treatment and deprivation period for ERβ (*F*(3,38) = 3.762, *p* = 0.014), but not ERα. ERβ mRNA expression was significantly decreased at 4, 8, and 12 weeks post-OVX in the vehicle treated animals compared to E_2_ treatment (Figure 8b, black line, #). Next, we ovariectomized aged (18 month old) Fischer 344 female rats and 1 week later administered either E_2_, or an ERα (PPT) or ERβ (DPN) selective agonist. We then measured the primary, precursor, and mature miR expression levels for each of our previously identified E_2_ regulated miRNAs using qRT-PCR (Figure 9). First, we analyzed the expression levels of the primary transcripts, which demonstrated that both PPT and DPN treatment significantly decreased the expression of pri-miR-7a, pri-miR-125a, pri-miR-181a, and pri-miR-495 compared to either vehicle or E_2_ treated animals (Figure 9a). These results were surprising given that no effect was observed with E_2_ treatment alone. Interestingly, let-7i primary transcript expression was the only miRNA to be significantly increased with DPN and PPT treatment (Figure 9a). By contrast, PPT significantly increased the expression of the precursor form of miR-181a (pre-miR-181a), but had no effect on the precursor forms for any of the other miRNAs (Figure 9b). Similarly, DPN significantly increased the precursor form of miR-495 (pre-miR-495), but not any other precursor miRNA (Figure 9b). Finally, DPN, but not PPT, mimicked the effects of E_2_ on the expression levels of mature let-7i, and miR-7a, which was consistent with our earlier observations after 1 week of E_2_ deprivation (Figure 9c).

The effects of PPT and DPN on miR-9 were particularly interesting. Our previously published observations, as well as replicated data herein, demonstrated that E_2_ significantly increased both the guide (miR-9) and passenger (miR-9-3p) strands of mature miR-9 after 1 week of E_2_ deprivation in aged animals. However, both PPT and DPN significantly decreased the pri-miR-9 and pre-miR-9 forms of this miRNA (Figure 9d, 9e). Notably, E_2_ tended to increase pri-miR-9, and decrease pre-miR-9, but neither effect was statistically significant. Finally, E_2_, PPT, and DPN all significantly increased mature miR-9 and miR-9-3p, but DPN increased each to a greater degree (Figure 9f, 9g).

## DISCUSSION

Our primary objective in these studies was to test the efficacy of acute E_2_ treatment, administered at different time points following the complete loss (via OVX) of ovarian hormones, on miRNA expression in the aged female brain. The results from this study demonstrated several novel findings. First, E_2_ treatment altered mature miRNA expression in the brain of aged animals after 1 week of ovarian hormone deprivation, which replicated our previously published findings, but this effect was lost following longer periods of ovarian hormone deprivation. These results are consistent with that of the Timing Hypothesis, which suggests that the efficacy of E_2_ action changes with advanced age and length of time postmenopause. Second, we showed that aging alone (i.e. ovarian intact animals) also had a significant effect on mature miRNA expression in many cases, and these are the first data to describe miRNA expression levels in the brains of animals at several time points of advanced age. Surprisingly, ovarian intact animals displayed a different miRNA expression profile then did the OVX+vehicle treated animals suggesting that these E_2_-regulated miRNAs are sensitive to ovarian factors in addition to E_2_, however, to our knowledge there is very little known about the regulation of miRNAs in the brain by other ovarian factors. Third, our results showed that E_2_ treatment selectively altered the expression of the primary and precursor transcripts of these same miRNAs, however those expression levels often did not correspond to the levels of their mature miRNA counterparts. Our interpretation of these data is that E_2_ can act at multiple levels along the miRNA biosynthesis pathway, and that E_2_ perhaps plays a role in the stabilization of mature miRNAs. Finally, using ER selective agonists we demonstrated that the effects of E_2_ on mature miRNA expression in the hypothalamus are likely mediated primarily through ERβ. Taken together these results extend our previous findings by suggesting a possible mechanism for the lack of therapeutic effectiveness for HT in post-menopausal women.

Aging has been shown to alter mature miRNA expression profiles in a variety tissues [34, 52, 53, 54, 55]. Our data (ovarian intact animals) also showed age-related changes in mature miRNA expression in the hypothalamus. Previous studies have shown that aging can differentially regulate distinct groups of miRNAs, suggesting that the miRNAs we tested in the present study could be part of a larger shift in miRNA expression in the brain. The mechanism by which aging alters miRNA expression is not currently known, though it has been shown that aging can alter post-transcriptional modifications of mature miRNAs thereby altering their stability [56]. Unexpectedly, the OVX + vehicle treated animals had a different aging miRNA expression pattern than did the ovarian intact animals, and the effects were distinct for each miRNA tested. Importantly, the circulating E_2_ levels were not different between the OVX+ vehicle and ovarian intact animals (Figure 1), suggesting that ovarian factors other than E_2_ contribute to mature miRNA expression levels in the aged female brain.

Our previous work showed that E_2_ differentially regulated the expression of mature miRNAs in the brains of young, compared with aged, female rats [46]. This current study extends those findings by focusing only on aged animals that were given E_2_ replacement at varying times post-OVX. Consistent with our earlier findings, E_2_ treatment significantly increased 5 out of 7 previously identified mature miRNAs after only a brief period of ovarian hormone deprivation (i.e. one week) in aged animals. It is important to note that these current studies examined mature miRNA expression only in the hypothalamus, whereas our previous findings were mostly derived from the hippocampus. Nevertheless, these data remained consistent with our earlier results in that there appears to be brain-region specific effects of E_2_ on miRNA expression. Notably, we observed no differences in mature miRNA expression between E_2_- and vehicle-treated animals following longer periods of ovarian hormone deprivation (i.e. 4, 8, and 12 weeks). These results are in-line with the concept of the Timing Hypothesis, which predicts a lack of E_2_ efficacy following longer periods of ovarian hormone deprivation. miRNAs are important regulators of mRNA translation and are predicted to impact the expression of more than 60% of all protein-coding genes [57]. The implication of these results suggests that a loss of E_2_-regulated miRNAs postmenopause could negatively impact multiple downstream target genes, although it remains unclear which specific downstream target genes these particular miRNAs regulate.

Despite the critical role of miRNAs in all physiological systems, little is known about the tissue-specific regulation of miRNA biosynthesis and processing. In general, global miRNA biogenesis follows a well-defined pathway beginning with the generation of a long primary transcript (pri-miR) and ending with a single-stranded 22 nt mature miRNA product [20, 21, 24, 25, 58]. There are several steps along this pathway that are potential points where unique tissue- and age-specific expression of individual miRNAs could occur. Estrogen receptors act primarily as transcription factors on various gene promoters, therefore, the most likely site of E_2_ regulation is at the transcriptional level of the primary miRNA form. Indeed, others have demonstrated that E_2_ regulates several pri-miRs through canonical estrogen response elements (EREs) in breast cancer cell models [49, 50, 59]. Therefore, we next measured the expression levels of the primary (pri-miR) and precursor (pre-miR) forms of our identified E_2_-regulated mature miRNAs at varying times post-OVX. Our prediction was that the expression levels of these primary transcripts would match that of the mature miRNA effector, however this was not what we observed. In fact, there was considerable discordance between the pri-, pre-, and mature forms for most of the miRNAs tested. The discordance observed could be the result of altered processing of these miRNAs. Post-transcriptional modifications to RNAs, including miRNAs, are common and important regulatory mechanisms, which can promote or inhibit their processing [22, 28, 29, 60]. These studies suggest that not only expression, but also sequence changes to the miRNA should be analyzed to more completely understand their regulation and function. There is little known about the temporal regulation of miRNA biosynthetic events, but these results suggest that E_2_ could regulate different steps along the pathway at different times. It also demonstrates a limitation of our studies in that each miRNA was measured at just one snapshot in time following E_2_ treatment (24 hours following the last injection). Nevertheless, the fact that E_2_ regulated all of the miRNAs forms (primary, precursor, or mature) at some time point indicates that E_2_ might also regulate some of the important key proteins involved in miRNA biogenesis and/or stabilization.

Mature single-stranded miRNA molecules in association with an AGO protein comprise the two core components of the RNA-induced silencing complex [61]. The mature miRNA molecule is formed after the cytoplasmic RNase III enzyme dicer cleaves the pre-miR to form a small miRNA duplex structure [20]. Only one strand of this duplex (leading strand) associates with AGO; the other strand (passenger strand) remains free, either to bind to RNAs and other proteins or be degraded [62, 63, 64]. In contrast to that description of miRNA formation, our data showed that E_2_ treatment not only increased the expression of mature miR-9 in aged females, but it also increased the expression of the miR-9a passenger strand, miR-9-3p (current study, and [46]). Because both strands of the miR-9 duplex are derived after dicer processing of the same precursor molecule, these results provide strong evidence that E_2_ can regulate miRNA expression at the level of molecular stabilization. Therefore, to determine if E_2_ regulates key components of miRNA biogenesis or stabilization we examined their mRNA and protein levels in our ovarian hormone deprivation paradigm. We observed that E_2_ treatment significantly increased drosha and dicer mRNA at 8 weeks post-OVX, however these changes were not mirrored by changes in protein levels at that same time point. One possibility is that we missed the window of detection, given that we measured both mRNA and protein at only one time point following E_2_ treatment (24 hours after the last injection). Moreover, the levels of mRNA and protein for these enzymes do not necessarily reflect changes in enzymatic activity. Importantly, the enzymatic activities are acutely sensitive to rapid changes in co-factor binding and posttranslational modifications [20, 22, 23, 58, 65], all of which are potential targets for E_2_ regulation. At this time, we are unaware of any enzymatic assay that has been developed for the detection of dicer or drosha activity either in cell line models or tissue, making this a very important future direction for research efforts. Overall, there were no significant changes due to age or treatment on the protein expression for any of the other key miRNA biogenesis or stabilization components tested. These results are consistent with our findings, and that of others, showing that E_2_ and age only regulate a very small subset of miRNAs [46, 51]. Therefore, E_2_ regulation of a major protein component in the miRNA biogenesis pathway is unanticipated, as that would predict a broad impact on a much greater number of miRNAs.

The results of the WHI studies prompted both basic and clinical scientists to reevaluate the role of E_2_ in the aging brain. Changes in the composition, number, or structure of estrogen receptors (ERα or ERβ) would be an obvious explanation for reduced E_2_ efficacy with age. Indeed, previously published studies have shown that both ERα and ERβ expression are altered with aging in specific nuclei of the hypothalamus, although the reported findings are somewhat contradictory with respect to ERβ [66, 67, 68]. Our data showed that there was a significant decrease in ERβ mRNA expression at 4, 8, and 12 weeks post-OVX (equivalent to 19, 20, and 21 months old, respectively) in the vehicle treated animals. However, treatment with the ERβ specific agonist DPN was equally effective as E_2_ treatment on mature miRNA expression, and there were no changes observed in ERα expression, suggesting that decreased ER availability is not a primary factor for the lack of E_2_ efficacy in the aged female brain. Another possibility is that the expression of fully functional ERβ splice variants is altered with age. For example, ERβ2 has a lower E_2_ binding affinity compared to the wild type ERβ. ERβ2 also has different transcriptional efficacy, its expression increases with age, and it was correlated with an increase in depressive behaviors in aged Sprague-Dawley female rats [69, 70, 71]. Our experiments were designed to detect the full-length wild-type ERβ, which would include detection of all known ERβ splice variants. Therefore, the ERβ mRNA expression levels that we observed would not differentiate between the relative amounts of ERβ2 at varying ages, and increases in these receptor splice variants could decrease E_2_ efficacy. Another possible explanation for reduced E_2_ efficacy over time could be that the expression and/or association of ER coregulatory proteins are altered with age. Indeed, we have previously demonstrated that the protein complexes associated with ERβ in the brain are altered by E_2_ in an age-dependent manner, despite no age-related changes in the expression of the cofactor proteins themselves [72]. These data indicate that altered ER and/or cofactor gene expression might not be good markers to assess the efficacy of E_2_ signaling.

Although the actions of E_2_ are mediated primarily through its two classical nuclear receptors, ERα and ERβ, recent evidence has emerged implicating E_2_ regulation of cell function through a variety of other intracellular signaling pathways, some of which are mediated by membrane-bound ER or G protein-coupled receptors. In these studies, we used a straightforward approach to try to identify whether the classical ERs were mediating the E_2_-induced regulation of mature miRNA expression in the aged brains following a brief period (1 week) of ovarian hormone deprivation. To that end, we took advantage of the recent development of the specific ERα and ERβ agonists, PPT (propylpyrazole-triol) and DPN (diarylpropionitrile), respectively. These agonists have been shown to be highly selective for their respective form of the receptor when used at the doses we administered [73, 74]. In general, our results indicated that the E_2_-induced regulation of mature miRNAs after one week of OVX is mediated by both receptors, though ERβ was able to specifically regulate let-7i and miR-7a independent of ERα. Interestingly, there were some instances, most notably at the primary (pri-miR) level, where PPT and DPN were equally efficacious and E_2_ had no apparent effect (see Figure 9). Notably, the high selectivity of PPT and DPN for their respective receptors indicates that they only bind and activate homodimer configurations of the receptors. Therefore, it is possible that the effects of E_2_ on miRNAs are mediated by ERα and ERβ heterodimers. Indeed, our data demonstrating that both DPN and PPT regulated pri-miRNA levels, yet there was no apparent effect of E_2_ on these same primary transcripts support that hypothesis. Further, it is also possible that there are changes in homo- and heterodimer formations that change with age and/or prolonged periods of E_2_ deprivation, suggesting that these ER selective agonists could be a useful therapeutic tool for postmenopausal women at specific ages.

## MATERIALS AND METHODS

### Ethics statement

All animal protocols were approved by the Institutional Animal Care and Use Committee at Loyola University Chicago, IACUC approval #2009018. Surgeries were performed under vaporized isoflurane anesthesia. Post-operation, animals were singly housed and provided with acetaminophen analgesic (122.7 mg/kg) in tap water for 3 days. All measures were taken to minimize pain and suffering.

### Animals

#### Experiment 1: Ovarian intact aged female rats

Fischer 344 rats were obtained from Charles River Laboratories (Wilmington, MA) at 18 months of age (*N* = 24). The animals were allowed to acclimate to the housing facility at Loyola for 7 days after arrival. Animals were housed two per cage and were allowed free access to standard rat chow and tap water. They were left undisturbed until euthanasia at 18, 19, 20, or 21 months of age.

#### Experiment 2: Acute treatment with vehicle/E_2_ at varying times post ovariectomy (OVX) in aged female rats

Female Fischer 344 rats were obtained from the National Institutes of Aging (NIA) colony (Taconic) at 18 months (*N* = 46) of age. The animals were allowed to acclimate to the housing facility at Loyola for 7 days after arrival. Animals were housed two per cage and were allowed free access to standard rat chow and tap water. Animals were OVX at 18 months of age after the acclimation period and then left undisturbed for 1, 4, 8, or 12 weeks (*N* = 11–12/age group) following OVX (Figure 1a). After the designated time the animals were given a subcutaneous injection of either safflower oil (vehicle; *N* = 5–6/age group) or 2.5 μg/kg 17β-estradiol (E_2_; *N* = 6/age group) dissolved in safflower oil once/day for 3 consecutive days. The animals were anesthetized and then euthanized by rapid decapitation 24 hours following the last E_2_ injection.

#### Experiment 3: Acute treatment of selective estrogen receptor agonists after 1 week post-OVX in aged female rats

Fischer 344 rats were obtained from Charles River Laboratories (Wilmington, MA) at 18 months of age (*N* = 14). Animals were housed two per cage and were allowed free access to standard rat chow and tap water. Animals were OVX at 18 months of age after the acclimation period, left undisturbed for 1 week and then were given a subcutaneous injection of either Diarylpropionitrile (DPN, Tocris, 1.0 mg/kg, *N* = 7) or 4,4′,4′’-(4-Propyl-[1*H*]-pyrazole-1,3,5-triyl)*tris*phenol (PPT, Tocris, 0.5 mg/kg, *N* = 7) once/day for 3 consecutive days. These doses of DPN and PPT have previously been shown to have physiological effects on cognitive function [73, 74].

### Ovariectomy

Animals were deeply anesthetized with vaporized isofluorane and bilaterally ovariectomized (OVX) as described previously [72]. Briefly, the ovary and distal end of the uterine horn were pulled from the body cavity through a 1 cm incision made through the skin and body wall. The uterine horn was clamped with a hemostat and ligated proximal to the clamp. The entire ovary and distal uterine horn were then removed. Animals were singly housed and provided with acetaminophen analgesic (122.7 mg/kg) in tap water for 3 days postoperative. During this time, animals were weighed once/day and their water intake was measured. Following 3 days of analgesia the animals were double-housed with their previous cage mate for the duration of the experiment.

### Blood collection and tissue processing

The animals were anesthetized using vaporized isofluorane and then euthanized by rapid decapitation 24 hours following the last treatment. Trunk blood was collected on ice into heparinized 10 ml round bottom tubes, centrifuged at 4000 RPM for 8 min., and plasma stored at −20°C until further processing. Brains were quickly removed, sagittally sectioned on ice into left and right hemispheres, and the whole hypothalamus was microdissected from the right side of the brain. The hypothalami were placed in separate microcentrifuge tubes containing QIAzol lysis reagent (Qiagen, Inc., Germantown, MD) for subsequent homogenization and RNA extraction. The left side of the brain was rapidly frozen in 2-methylbutane, sectioned at 200 μm on a freezing microtome, and the whole hypothalamus (−0.26 to −4.52 relative of bregma, according to Paxinos and Watson, Rat Brain Atlas, Fourth edition), was microdissected using a Palkovit's brain punch tool (1.0 mm, Stoelting, Inc., Wood Dale, IL). Tissue punches were placed in a microcentrifuge tube on dry ice and stored at −80°C.

### 17β-estradiol plasma concentrations

The plasma samples first underwent a liquid-liquid extraction using diethyl ether to eliminate interfering compounds in the plasma as previously described [75]. Following diethyl ether extraction, samples were reconstituted using sample buffer contained in the 17β-estradiol high sensitivity ELISA kit (Enzo Life Sciences, Cat. No. AD 901 174), which was used to determine concentration of circulating E_2_ levels. Absorbance was measured on a BioTek (Winooski, VT) Synergy HT plate reader. The circulating plasma E_2_ concentrations in the E_2_ and vehicle animals were 56.68 (+/− 20.29) and 36.10 (+/− 9.70) pg/mL respectively (Figure 1b). These E_2_ levels were consistent with physiological levels observed during late diestrous/early proestrous and with levels achieved in postmenopausal women following HT [76]. The intra- and inter-assay %CV was 4.23 and 6.85 pg/ml respectively.

### RNA isolation

Total RNA was isolated from the whole right hypothalamus using the miRNeasy Mini Kit (Qiagen, Inc., Germantown, MD) according to manufacturer's instructions. All RNA samples were quantified and analyzed for quality using Nanodrop spectrophotometry and visualization of the RNA on 1.5% agarose gel.

### Precursor and mature miRNA cDNA synthesis

Total RNA (1.0 μg) was used to reverse transcribe miRNA using NCode™ VILO™ miRNA cDNA synthesis kit (Life Technologies, Carlsbad, CA) to assay for mature miRNA expression. To assay for the primary and precursor miRNA, we utilized a miRNA specific reverse transcription method described previously [77]. Briefly, 2.0 μg of total RNA was added to 1.5 μl of primer cocktail containing 10 μM of antisense primers (Supplementary Table S1). The reaction mixture was incubated at 80°C for 5 minutes, 60°C for 5 minutes, and then finally allowed to cool to room temperature. Reagents from the ThermoScript™ RT-PCR System (Life Technologies, Carlsbad, CA) were then added for a final volume of 20 μl. The reactions were incubated at 60°C for 45 minutes and then 85°C for 5 minutes. 1.0 μl of RNase H was added to each reaction and then incubated at 37°C for 20 minutes.

### Messenger RNA(mRNA) cDNA synthesis

Total RNA (2.0 μg) was reverse transcribed using the High Capacity cDNA Reverse Transcriptase Kit (Applied Biosystems, Foster City, CA).

### Quantitative real-time qRT-PCR

miRNA and mRNA qRT-PCR was performed with Fast Start Universal SYBR Green Master Mix (Roche-Genentech, San Francisco, CA) on an Eppendorf Realplex4. Forward primers for specific mature miRNAs were designed as described in the Ncode™ miRNA First-Strand cDNA synthesis kit handbook (Life Technologies, Carlsbad, CA) and using miRBase 18 as a sequence reference (Supplementary Table S1). Primary and precursor miRNA primers were designed as described previously (Supplementary Table S1) [77]. Primers for mRNAs were obtained from previous published studies [27, 78] (Supplementary Table S2). 18s rRNA and HPRT were used as a loading control and to normalize the data for analysis for miRNA and mRNA qRT-PCR respectively. The following program was used for miRNA qRT-PCR: 1) 95°C for 10 minutes, 2) 95°C for 20 seconds, 3) 59°C for 20 seconds, 4) 72°C for 12 sec, and melting curve analysis (Supplementary Table S1). The following program was used for mRNA qRT-PCR: 1) 95°C for 10 minutes, 2) 95°C for 30 seconds, 3) 59°C for 30 seconds, 4) 72°C for 30 sec, and melting curve analysis (Supplementary Table S2). ERβ mRNA expression was analyzed using Taqman Gene Expression Assay (Rn01527840_m1) with a custom FAM probe for ERβ (CAAGAAAATCCCTGGCTTTGTGGAG) (Supplementary Table S2). The following program was used for Taqman RT-PCR: 1) 95°C for 10 minutes, 2) 95°C for 15 seconds, and 3) 60°C for 60 seconds. miRNA and mRNA expression was analyzed using the ΔΔCt method as described previously [79].

### Western blots

Protein lysate from hypothalamic tissue punches was isolated using T-PER reagent (Thermo Scientific, Waltham, MA). Briefly, tissue punches were homogenized in 500 μl of T-PER reagent with 1x cOmplete mini EDTA-free protease inhibitor cocktail (Roche-Genentech, San Francisco, CA) with a motorized mortar. Tissue lysate was centrifuged at 10,000 × g for 5 minutes and the supernatant was collected into a separate microfuge tube. Protein samples were then concentrated using a methanol-chloroform extraction as described previously [80]. Total protein lysate (60 μg) was dissolved and boiled at 95°C for 5 minutes in a 1x reducing sample buffer (Thermo Scientific, Waltham, MA). Protein samples were resolved on a gradient (4–10%) polyacrylamide gel at 90V for 20 minutes and then 120V for 60 minutes using Bio-Rad Mini-Protean 3 system. Separated proteins were then transferred to PVDF Immobilon-P membranes (Millipore, Billerica, MA) at 100V for two hours. Membranes were blocked with 5% BSA with 1x TBST (0.1% tween) for 1 hour. Membranes were then incubated with the indicated primary antibody overnight with constant shaking at 4°C (Supplementary Table S3). The primary antibody was removed and the membranes were rinsed three times with 1x TBST (0.1% tween). Membranes were then incubated with 1:5000 goat anti-rabbit HRP secondary antibody (Santa Cruz Biotechnology, Dallas, Texas) in 5% BSA with 1x TBST (0.1% tween) for two hours with shaking at room temperature. Membranes were then rinsed with 1x TBST (0.1% tween) three times and then developed using Super Signal West Pico Chemiluminescent substrate (Thermo Scientific, Waltham, MA). Blots were visualized using the Bio-Rad Chemi-doc stations. Densitometry analysis was performed using Image Lab software (Bio-Rad Laboratories, Des Plaines, IL). Membranes were then stripped using a mild stripping buffer (Abcam, Cambridge, England) and re-blotted with another primary antibody. Beta actin expression was used as the loading control and to normalize the data for analysis.

### Statistics

Expression of miRNAs and mRNAs in the ovarian hormone deprivation paradigm were analyzed by two-way ANOVA with age and treatment as factors. A significant interaction between age and treatment was followed by a Tukey's post hoc test to determine statistically significant differences (*p* < 0.05) between groups. A separate Tukey's post-hoc test was performed within groups that showed a statistically significant main effect of age and/or treatment. When no significant interaction was observed, the two factors were considered independent and Student's *T*-tests were used to analyze between treatments within a single time point. A one-way ANOVA with treatment as the main factor, followed by Tukey's posthoc test, was used to determine significant differences of E_2_, DPN, and PPT. All data are presented as mean ± SEM. Statistical significance was noted when *p* < 0.05.

## SUPPLEMENTARY MATERIALS TABLES


