# Supplementary material for: Prolonged ovarian hormone deprivation alters the effects of 17β-estradiol on microRNA expression in the aged female rat hypothalamus
Source: Oncotarget. 2015 Oct 9;6(35):36965–83. doi: 10.18632/oncotarget.5433 (PMC4741909; doi:10.18632/oncotarget.5433)
Supplement: Supplementary file 1 [file oncotarget-06-36965-s001.pdf]

SUPPLEMENTARY TABLES

Supplementary Table S1: miRNA real time RT-PCR primer sequences and annealing temperatures

Name	Forward (5′-> 3′)	Reverse (5′-> 3′)	Annealing Temperature (°C)
let-7i	CGCGTGAGGTAGTAGTTTGTGCTGTT	Ncode VILO universal reverse primer	59
miR-7a	GCGCTGGAAGACTAGTGATTTTGTTGT	Same as above	59
miR-9	CGCGTGTTTGGTTATCTAGCTGTATG	Same as above	59
miR-9-3p	CGCGATAAAGCTAGATAACCGAAAG	Same as above	59
miR-125a	CGTCCCTGAGACCCTTTAACCTGTGA	Same as above	59
miR-181a	CGAACATTCAACGCTGTCGGTGAGT	Same as above	59
miR-495	CAGGCCAAGACAGTATCTCCCTCA	Same as above	59
Pre-let-7i	CGCGTGAGGTAGTAGTTTGTGCTGTT	AGCAAGGCAGTAGCTTGCGCAG	59
Pre-mir-7a	GCGCTGGAAGACTAGTGATTTTGTTGT	ATGGCAGACTGTGATTTGTTGT	61
Pre-mir-9	CGCGTGTTTGGTTATCTAGCTGTATG	ACTTTCGGTTATCTAGCTTTAT	61
Pre-mir-125a	CGTCCCTGAGACCCTTTAACCTGTGA	GGCTCCCAAGAACCT CACCTGT	65
Pre-mir-181a	CGAACATTCAACGCTGTCGGTGAGT	GGTACAATCAAC GGTCGATGGT	65
Pre-mir-495	AAGAAGTTGCCCATTTATTTTTCGC	AAGAAGTGCACCATGTTTGTTT	61
Pri-let-7i	ACCATGGCCCTGGCTGAGGTA	AGCAAGGCAGTAGCTTGCGCAG	63.4
Pri-mir-7a	CATTTCTCTGGTGAAAACTGCTGCC	ATGGCAGACTGTGATTTGTTGT	65.1
Pri-mir-9	AGGCTGCGTGGAAGAGGAG	ACTTTCGGTTATCTAGCTTTAT	63.4
Pri-mir-125a	CTTTTTCTGTCACTCTTCCCTGCTC	GGCTCCCAAGAACCT CACCTGT	65
Pri-mir-181a	GAAGGAATCCCGCTTCTTTTCTTCC	GGTACAATCAAC GGTCGATGGT	65
Pri-mir-495	TAACACTCAGACACACTGCTGGAAC	AAGAAGTGCACCATGTTTGTTT	62.3

Supplementary Table S2: mRNA real time RT-PCR primer sequences and annealing temperatures

Name	Forward (5′-> 3′)	Reverse (5′-> 3′)	Annealing Temperature (°C)
Drosha	GAAGTCACCGTGGAGCTGAGTA	ATCATTGCATGCTGACAGACATC	55
DGCR8	TCAAGGTCCGCCCTGTTTAT	GAGGCACCAAAAGGCTCACTT	59
Exportin 5	GACGCAGAACATGGAAAGAATCT	TGTCTTCATTTGTTGGTACTTGTTTAC	59
Dicer	GGGAAAGTCTGCAGAACAAAC	GGCTGTCTGAGCTCTTAGTTC	55
Argonaute 2	CCTGAGAAATGCCCTCGGAGAGTGA	GACCTCCAGCTCCACCTTGTCCCTG	59
PAPD4	ACAGGGTTGTCTACGCCGCC	CGCGGGCGTGTTAAGTTGGG	60
XRN2	TCGAGGAGGGCGACAGGGAT	GGGCGGTGGCAAAGGGTACT	60
ERα	AATTCTGACAATCGACGCCAG	GTGCTTCAACATTCTCCCTCCTC	57
ERβ	GAGAGACACTGAAGAGGAAGC	TCACGGAACCTTGACGTCGTC	60

Supplementary Table S3: Western blot antibody information

Primary Antibody	Company	Catalog #	Dilution used
Drosha	Santa Cruz	Sc-33778	1:300
DGCR8	Novus Biologicals	NBP1–57261	1:1000
XPO5	Abcam	ab129006	1:1000
Dicer	Santa Cruz	Sc-30226	1:150
Argonaute 2	Cell Signaling	2897S	1:200
β-actin	Cell Signaling	4970S	1:1000
